# Timing of systemic resistance induced by local exogenous ABA application within clonal network of stoloniferous herb *Centella asiatica* subjected to low water availability

**DOI:** 10.3389/fpls.2023.1324460

**Published:** 2024-01-10

**Authors:** Su-Juan Duan, Gui-Jia Sun, Yi Dan, Jie Deng, Dong-Wei Yu, Qin Wei, Chang-Fan Chen, Jie Jiang, Xue-Mei Wang, Ting-Ju Ren, Yong-Mei Liao, Jin-Song Chen

**Affiliations:** ^1^ Key Laboratory of Southwest China Wildlife Resources Conservation, China West Normal University, Ministry of Education, Nanchong, China; ^2^ College of Life Science, Sichuan Normal University, Chengdu, China; ^3^ Shi Fang Municipal People’s Government Office, Deyang, China; ^4^ State Key Laboratory of Grassland Agro-ecosystems, College of Pastoral Agricultural Science and Technology, Lanzhou University, Lanzhou, China

**Keywords:** clonal integration, resistance activation, resistance delay, chlorophyll fluorescence, photosynthetic parameters

## Abstract

Resistance traits of plants can be activated both at the damaged site and undamaged parts. Systemic resistance induced by local exogenous abscisic acid (ABA) application alleviated negative effect of low water availability on growth performance of clonal plant. However, timing of systemic resistance was poorly understood. Timing of systemic resistance refers to its activation and decay time within clonal network. Clonal fragment of *Centella asiatica* with four successive ramets (including first-oldest, second-older, third-old and fourth-young ramets) subjected to low water availability (20% soil moisture content) was used to explore effects of local exogenous ABA application on the timing of resistance activation and decay. Systemic resistance activated by local exogenous ABA application after 4 days remained at least 28 days. Compared with control, biomass accumulation of whole clonal fragment, root biomass and ratio of belowground to aboveground biomass significantly increased by local exogenous ABA application after 28 days. It is suggested that rapid activation and delay of resistance response induced by local exogenous ABA application within clonal network may improve fitness of clonal plant subjected to abiotic stress.

## 1 Introduction

Non-resource substances (such as defense or stress signal) can be transmitted or shared between interconnected ramets of clonal plant as well as resource substances (Stuefer et al., 2005; Jelinkova et al., 2012; Liu et al., 2015). With the increase of foliar tannin content, growth performance of interconnected young ramets was improved by local herbivory on old ramets of stoloniferous herb *Trifolium repens* (Gomez et al., 2008). Similarly, damage of caterpillar *Gynaephora rnenyuanensis* herbivory on young ramets of rhizomatous sedge *Carex alrofusca* was significantly alleviated by local application of jasmonic-acid to interconnected old ramets (Chen et al., 2011). Systemic defense or resistance within clonal networks induced by transportation or sharing of non-resource substances (such as defense or stress signal) may be very important for improving fitness of clonal plant subjected to biotic or abiotic stress (Gomez et al., 2007; Koubek and Herben, 2007; Sharifi and Ryu, 2021).

Systemic defense of soybean (*Glycine max*) induced by Mexican bean beetle (*Epilachnavarivestis*) herbivory after damage by 3 days gradually decayed by 15 days after damage (Underwood, 1998). With enhanced expression of defense-related genes, phytohormone concentration of leaf tissue (such as jasmonic acid and linolenic acid) significantly increased when leaf of hybrid poplar saplings was exposed to volatile compounds (*cis*-3-hexenyl acetate) for 72-96 hours (Frost et al., 2008). Foliar palatability of stoloniferous herb *T. repens* decreased local herbivory attack of *Mamestra brassicae* larvae after damage by 38-51 hours, which lasted for 28 days at least among interconnected undamaged ramets (Gomez et al., 2010). Therefore, rapid activation and delay of systemic defense induced by local herbivory within clonal network may improve fitness of clonal plant subjected to abiotic stress.

Exposure to volatile organic compounds (bacterial volatile blends from *Bacillus subtilis* GB03 and *Bacillus amyloliquefaciens* IN937a) from rhizobacteria for as little as 4 days was sufficient to activate induced systemic resistance in *Arabidopsis* seedlings (Ryu et al., 2004). Melatonin application improved the activity of antioxidant enzymes [APX (ascorbate peroxidase), CAT (catalase), DHAR (dehydroascorbate reductase), GST (glutathione S-transferase), GR (glutathione reductase), MDHAR (monodehydroascorbate reductase), POD (peroxidase), and SOD (superoxide dismutase)] and their relative genes expression when tomato seedlings were subjected to drought stress (Altaf et al., 2022). With systemic resistance activation, oxidative stress (O_2_^•−^ production rate and MDA content) in the leaf of the old, mature and young ramets was significantly alleviated by exogenous ABA application to the oldest ramets of stoloniferous herb *C. asiatica* subjected to low water availability (Wei et al., 2019). However, timing of systemic resistance (activation and decay time) induced among interconnected ramets was poorly understood.

Production of highly oxidizing ROS immediately affected photosynthesis when the plant was subjected to biotic or abiotic stress (Singh and Thakur, 2018; Qamer et al., 2021; Sachdev et al., 2021). Activation of MPK3/MPK6 can rapidly alter the expression of photosynthesis-related genes and inhibit photosynthesis when *Arabidopsis thaliana* was subjected to *Pseudomonas syringae* infection (Su et al., 2018). Young leaves of *A. thaliana* acclimate better to the onset of water deficit by dissipating the excess excitation energy by NPQ (Sperdouli and Moustakas, 2011). Therefore, plant subject to biotic or abiotic stress can also be evaluated by measuring photosynthetic efficiency such as maximum quantum yield of PSII (F_v_/F_m_), effective PSII quantum yield (ΦPSII), photochemical quenching (qP) and non-photochemical quenching (NPQ)(Corcuera et al., 2011; Lucas et al., 2014; Chen et al., 2016; He et al., 2018).

**Figure 1:**
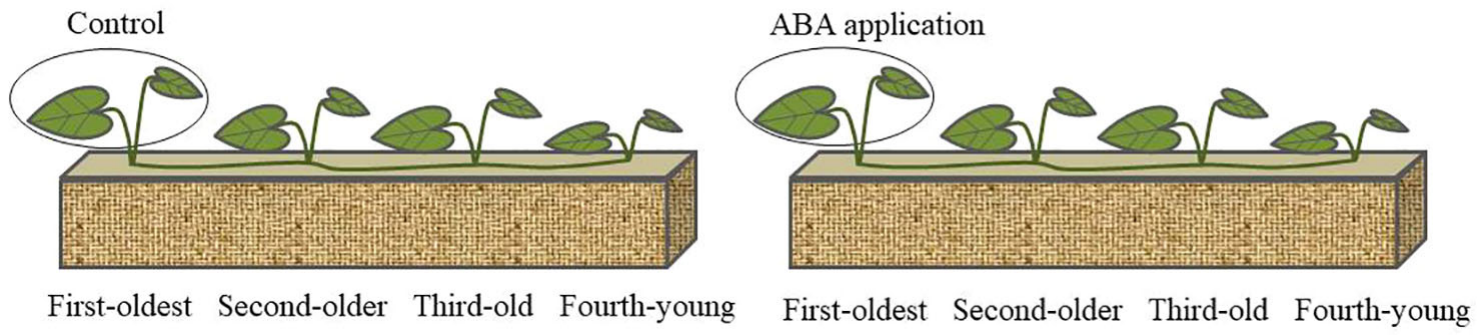
Schematic representation of the experimental design. 5 mL ABA solution (0.1mM) was applied to the first-oldest ramets; Same volume distilled water was employed as control.

The phytohormone abscisic acid (ABA) is a key endogenous messenger in plants’ responses to biotic and abiotic stresses such as various pathogens, heat, drought and high salinity (Yoshida et al., 2010; Osakabe et al., 2014; Lievens et al., 2017; Hu et al., 2018). It is rapid accumulation in response to stresses and mediation of many stress responses that help plant survival over the stresses (Sreenivasulu et al., 2012). Abscisic acid (ABA) as a stress hormone in plant responses to water shortage were well documented (Zhang et al., 2006; Zou et al., 2010; Yoshida et al., 2019). A greenhouse experiment with local application of exogenous ABA was conducted to investigate the timing of systematic resistance within clonal networks (**Figure 1**). We focused on (1) activation time of systemic resistance by local exogenous ABA application within clonal network of *C. asiatica*; (2) delay time of systemic resistance within clonal network after local exogenous ABA application. This research will help us to realize the mechanism for growth and fitness of clonal plant subjected to environmental stress.

## 2 Materials and methods

### 2.1 Plant material

As a perennial stoloniferous herb, *C. asiatica* was widely distributed in woodlands, forests edge, damp grass and roadsides or creeks. It usually takes root on each node of stolon when in contact with a moist substratum, forming a sympodial network above the ground (Li et al., 2018).

Clonal fragments of *C. asiatica* were collected from a forest edge, located in Chengdu, Sichuan province, China (30°05′~31°26′N; 102°54′~104°53′E). Each clonal fragment comprise**s** four rooted ramets with different age (first-oldest, second-older, third-old and fourth-young ramets).

### 2.2 Experimental design

The container (dimensions: 10 cm × 8.5 cm × 15 cm) separated into 4 equal pots was used for the experiment. On 18 October 2021, four successive ramets of each clonal fragment were planted into the pots respectively. The pots were filled with substrate in a 3:1 mixture of humus soil and sand. 0.2 g Peters Professional (20% N, 20% P, 20% K; The Scotts Company, LLC., Marysville, OH, USA) was added to each pot at the beginning of experiment. The volumetric soil moisture content of each pot was maintained at 20% (volume of water present/the total volume). In the everyday morning (9:00-11:00 h), all pots were measured with a portable soil moisture meter (TDR-300, Spectrum, USA) and watered to maintain corresponding soil moisture. During the experiment, the mean temperature was 28.5 ± 1.4°C, and light intensity was equivalent to approx. 90% of full daylight outside the greenhouse (minimum and maximum photosynthetic photon flux density in the greenhouse was 136.2 and 325.1 μmol m ^-2^ s^-1^ respectively).

In the experiment, 5 ml of 0.1 mM ABA solution was applied to fully unfolded leaves of the first-oldest ramets and the same volume of distilled water was used as control. ABA dosage was based on a previous study (Wei et al., 2019). Neighboring sibling ramets were shielded from spray with a piece of plastic. Then, the first-oldest ramets were sealed in a transparent plastic bag until dry. The chlorophyll fluorescence parameters and photosynthetic parameters were measured at 1, 4, 7 and14 days after ABA application. The experiment lasted for 28 days. There were seven replicates for per treatment.

### 2.3 Measurement of chlorophyll fluorescence parameters

Chlorophyll fluorescence measurements were carried out using a portable, modulated fluorescence monitoring system (FMS-2, Hansatech Instruments Ltd., UK) on fully expanded leaves. The minimum fluorescence (F_0_) was determined using a measuring beam of 0.2 μmol m^-2^ s^-1^ intensity after 30 min of dark adaptation. Following a dark-adapted state, a saturation pulse (1 s white light of 7,500 μmol m^-2^ s^-1^ intensity) was used to obtain the maximum fluorescence (F_m_). Light-induced changes in chlorophyll fluorescence following actinic illumination (300 μmol m^-2^ s^-1^) were recorded prior to the measurement of F′_o_ (minimum fluorescence in light-saturated state), F′_m_ (maximum fluorescence in light-saturated state) and F_s_ (steady-state fluorescence in the light-saturated state). The maximum quantum yield of PSII (F_v_/F_m_), the effective PSII quantum yield (ΦPSII), the photochemical quenching (qP) and non-photochemical quenching (NPQ) were calculated using (F_m_-F_0_)/F_m_, (F′_m_-F_s_)/F′_m_ (Genty et al., 1989), (F′_m_-F_s_)/(F′_m_-F′_0_) and (F_m_-F′_m_)/F′_m_ respectively (Turan and Ekmekçi, 2010).

### 2.4 Measurement of photosynthetic parameters

Photosynthetic parameters were made by a portable photosynthesis system GFS-3000 (Heinz Walz GmbH, Effeltrich, Germany). The measurement was conducted on the fully expanded mature leaves at a temperature of 25°C, photosynthetic photon flux density of 400 μmol·m^-2^·s^-1^ and CO_2_ concentration of 400 μmol·mol^-1^. Net photosynthetic rate (P_n_) and stomatal conductance (G_s_) were recorded when gas exchange had equilibrated (taken to be when the coefficient of variation for external CO_2_ partial pressure between the sample and reference analysis was below 0.3%).

### 2.5 Measurement of biomass characteristics of whole clonal fragment

Clonal fragments were separated into root, leaf and stolon and oven-dried to constant weight at 70°C for 72 h. Leaf and stolon biomass, root biomass, and biomass accumulation of whole clonal fragment were determined. Ratio of belowground to aboveground biomass was counted in whole clonal fragments (He et al., 2021).

### 2.6 Statistical analysis

The chlorophyll fluorescence parameters and photosynthetic parameters were analyzed by two-way repeated-measures (ANOVA). Two-way analysis of variance (ANOVA) was employed to investigate the leaf and stolon biomass, root biomass, ratio of belowground to aboveground biomass and biomass accumulation of whole clonal fragment. All analyses were conducted with SPSS 24.0 software (SPSS Inc.).

## 3 Result

### 3.1 Chlorophyll fluorescence parameters

**Figure 2:**
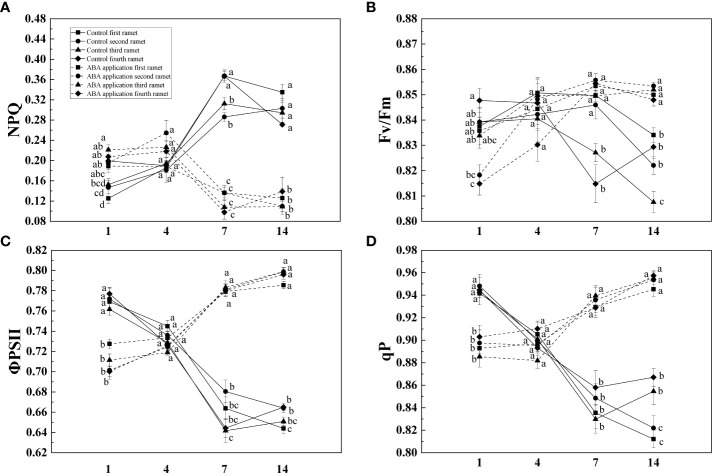
NPQ **(A)**, F_v_/F_m_ **(B)**, ΦPSII **(C)** and qP **(D)** of interconnected ramets (including first-oldest, second-older, third-old and fourth-young ramet) 1, 4, 7 and 14 days after local exogenous ABA application. Same letters (at the same day) mean that they are not significantly different (*P* > 0.05).

Compared with control, NPQ of four interconnected ramets was decreased by local exogenous ABA application after 1 day (**Table 1**, **Figure 2**). Opposite pattern was observed in ΦPSII, F_v_/F_m_ and qP (**Table 1**, **Figure 2**). After 4 days, significant difference was not observed between ΦPSII, F_v_/F_m_, qP and NPQ of four interconnected ramets subjected to local exogenous ABA application and those of control (**Table 1**, **Figure 2**). After 7 and 14 days, ΦPSII, F_v_/F_m_, and qP of four interconnected ramets subjected to local exogenous ABA application were significantly greater than those of control as well as significant decrease of NPQ (**Table 1**, **Figure 2**). From 7 to 14 days, significant effects of local exogenous ABA application on ΦPSII of four interconnected ramets were detected. However, significant effects of local exogenous ABA application on F_v_/F_m_, qP and NPQ of four interconnected ramets were not detected (**Table 1**, **Figure 2**).

**Table 1:** Results of two-way repeated-measures analysis of variance, with ‘exogenous ABA application’ and ‘ramet age’ as between-subject effects for differences in chlorophyll fluorescence parameters and photosynthetic parameters among interconnected ramets.

Effects	df	ΦPSII		qP		NPQ		F_v_/F_m_		Stomatal conductance		Photosynthesis	
		F	*P*	F	*P*	F	*P*	F	*P*	F	*P*	F	*P*
Between-subject effects													
Exogenous ABA application (A)	1	738.284	**0.001**	171.218	**0.001**	208.048	**0.001**	171.218	**0.001**	342.657	**0.001**	398.312	**0.001**
Ramet age (R)	3	4.11	**0.017**	3.479	**0.032**	0.644	0.582	3.479	**0.032**	1.596	0.23	3.455	**0.042**
A × R	3	4.964	**0.008**	0.667	0.58	2.726	*0.066*	0.667	0.58	2.273	0.119	2.622	*0.068*
Within subject effects													
Time (T)	3	9.438	**0.001**	12.033	**0.001**	21.315	**0.001**	12.033	**0.001**	116.92	**0.001**	317.862	**0.001**
T × A	3	542.905	**0.001**	37.357	**0.001**	204.263	**0.001**	37.357	**0.001**	228.454	**0.001**	612.769	**0.001**
T × R	9	2.391	**0.02**	2.807	*0.07*	2.55	**0.013**	2.807	**0.007**	0.905	*0.0529*	3.77	**0.001**
T × A × R	9	1.446	0.185	3.73	**0.001**	2.634	**0.011**	3.73	**0.00**1	1.198	**0.0318**	2.534	**0.018**

Values are in bold when *P* < 0.05, and in italic when 0.05 < *P*< 0.1.

### 3.2 Photosynthetic parameters

**Figure 3:**
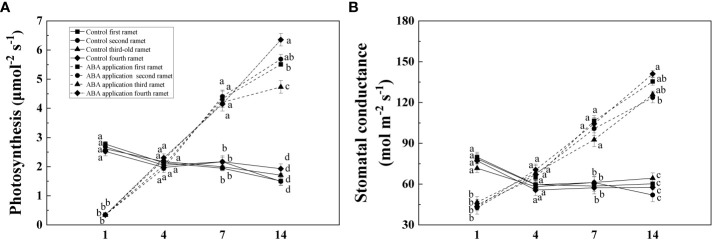
Net photosynthetic rate **(A)** and stomatal conductance **(B)** of interconnected ramets (including first-oldest, second-older, third-old and fourth-young ramet) 1, 4, 7 and 14 days after local exogenous ABA application. Same letters (at the same day) mean that they are not significantly different (*P* > 0.05).

Compared with control, P_n_ and G_s_ of four interconnected ramets were significantly decreased by local exogenous ABA application after 1 day (**Table 1**, **Figure 3**). After 4 days, significant difference was not observed between P_n_ and G_s_ of four interconnected ramets subjected to local exogenous ABA application and those of control (**Table 1**, **Figure 3**). After 7and 14 days, P_n_ and G_s_ of four interconnected ramets subjected to local exogenous ABA application were significantly greater than those of control (**Table 1**, **Figure 3**). G_s_ of fourth-young ramets was significantly greater than second-older ramets by local exogenous ABA applications after 14 days (**Table 1**, **Figure 3**). Meanwhile, P_n_ of fourth-young ramets was significantly greater than those of the first-oldest and third-old ramets (**Table 1**, **Figure 3**). From 7 to 14 days, significant effects of local exogenous ABA application on P_n_ and G_s_ of four interconnected ramets were detected (**Table 1**, **Figure 3**).

### 3.3 Biomass accumulation

**Figure 4:**
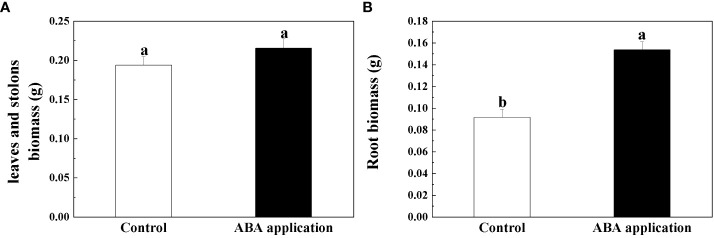
Leaf and stolon biomass **(A)** and Root biomass **(B)** of interconnected ramets after 28 days; Open bars, control; closed bars, exogenous ABA application. Error bars indicate ± s.e. for 10 replicates. Bars with the same lower case letters are not significantly different (*P* > 0.05).

**Figure 5:**
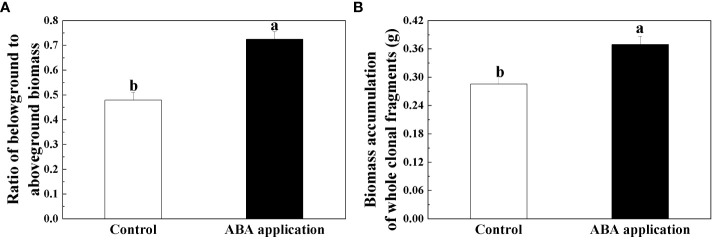
Ratio of belowground to aboveground biomass **(A)** and Biomass accumulation of whole clonal fragments **(B)** after 28 days; Open bars, control; closed bars, exogenous ABA application. Error bars indicate ± s.e. for 10 replicates. Bars with the same lower case letters are not significantly different (*P* > 0.05).

Root biomass, ratio of belowground to aboveground biomass and biomass accumulation of whole clonal fragments were significantly increased by local exogenous ABA application after 28 days (**Table 2**, **Figures 4B**, **5A, B**). However, similar patterns were not observed in leaf and stolon biomass (**Table 2**, **Figure 4A**).

**Table 2:** Two-way analysis of variance (ANOVA) for effects of ABA application, ramet age and their interaction on leaf and stolon biomass, root biomass, biomass accumulation of whole clonal fragments and ratio of belowground to aboveground biomass.

Effects	df	leaf and stolon biomass		root biomass		biomass accumulation of whole clonal fragments		ratio of belowground to aboveground biomass	
		F	*P*	F	*P*	F	*P*	F	*P*
Exogenous ABA application (A)	1	1.829	0.189	38.731	**0.000**	11.914	**0.002**	53.017	**0.000**
Ramet age (R)	3	1.461	0.250	1.205	0.329	0.206	0.891	8.628	**0.000**
A×R	3	0.623	0.607	1.710	0.192	1.117	0.362	0.792	0.511

Values are in bold when *P* < 0.05, and in italic when 0.05 < *P*< 0.1.

## 4 Discussion

Stomatal closure resulting from exogenous ABA application reduced water loss of wheat (Travaglia et al., 2010). In this study, with the stomatal closure, foliar net photosynthetic rate of four interconnected ramets significantly decreased by local exogenous ABA application after 1 day. By altering the kinetics of de-epoxidation of the xanthophyll cycle, exogenous ABA application incurred increase of NPQ in cabbage (*Brassica campestris*) and rice (*Oryza sativa L*) (Zhu et al., 2011). With its inhibition on photochemical activity, increase of NPQ implied that more light energy was used for heat dissipation to avoid damage to photosystem II of four interconnected ramets (Wilson and Ruban, 2020). Meanwhile, photoinhibition (decrease of F_v_/F_m_, ΦPSII and qP) was induced by abscisic acid (ABA) application after 1 day when clonal fragments of *C. asiatica* subjected to low water availability. Similar patterns were observed in the study that exogenous ABA application resulted in decrease of F_v_/F_m_, ΦPSII and qP of maize subjected high light intensity (1500 μmol m ^-2^ s ^-1^) (Jia and Lu, 2003).

ΦPSII and qP of sugarcane subjected to drought treatment were increased by the exogenous ABA application after 3 days and remained at least 7 days (Srivastava et al., 2009). Selenium (Se) application can alleviate oxidative stress in the chloroplasts to increase F_v_/F_m_ when potato (*Solanum tuberosum* L.) was subjected to photooxidative stress (Turakainen et al., 2008). In this study, chlorophyll fluorescence and photosynthesis of four interconnected ramets were restored by local exogenous ABA application after 4 days. Altogether, the recovery of chlorophyll fluorescence and photosynthesis capacity are interpreted as activation of systemic resistance. With the exogenous ABA application, root growth was improved when *Arabidopsis* seedlings was subjected to low water availability (Miao et al., 2021). Exogenous ABA application significantly increased root/shoot ratio of *Malus sieversii* and *Malus hupehensis* seedlings subjected to low water availability. Similar pattern was observed in our experiment (Ma et al., 2008). Biomass accumulation significantly increased by local exogenous ABA application when wheat was subjected to low water availability (Kaur and Asthir, 2020). The positive effects on growth performance of whole clonal fragments were observed by local exogenous ABA application after 28 days. We tentatively suggested that defense induction persisted for at least 28 days.

Systemic resistance may give priority to protection of youngest tissues (Chen et al., 2011). Young ramets were the most valuable tissues for growth and fitness within clonal networks (Stuefer et al., 2005; Gomez and Stuefer, 2006). The optimal defense theory predicts that plant tissues with a high contribution to fitness should be better protected than other plant tissues (Hunziker et al., 2021). In the experiment, our study was consistent with previous study that compared with the old and mature ramets, foliar antioxidant capacity of young ramets was significantly higher and oxidative stress was significantly lower when exogenous ABA application to the oldest ramets (Wei et al., 2019). It is suggested that the protection of young ramets may confer clonal plants with considerable benefits in adapting to environmental stress.

Our study implies that rapid activation and delay of resistance response induced by local exogenous ABA application within clonal network may improve fitness of clonal plant subjected to abiotic stress. Benefit of systemic resistance will depend on the absence or presence of subsequent environmental stress (van Hulten et al., 2006). In the future, more studies are needed to understand the generality and ecological advantages afforded by systematic resistance within clonal network.

## Data availability statement

The original contributions presented in the study are included in the article/supplementary material. Further inquiries can be directed to the corresponding authors.

## Author contributions

S-JD: Writing – original draft, Data curation, Investigation, Methodology, Conceptualization, Formal Analysis, Software, Validation, Visualization. G-JS: Data curation, Methodology, Investigation, Formal Analysis, Writing – original draft. YD: Investigation, Methodology, Data curation, Writing – original draft. JD: Methodology, Data curation, Investigation, Writing – original draft. D-WY: Methodology, Data curation, Writing – original draft. QW: Methodology, Data curation, Writing – original draft. C-FC: Investigation, Writing – original draft. JJ: Investigation, Writing – original draft. X-MW: Investigation, Writing – original draft. T-JR: Investigation, Writing – original draft. Y-ML: Writing – review & editing, Supervision, Validation. J-SC: Writing – review & editing, Supervision, Validation.

## References

[B1] Altaf M. A. Shahid R. Ren M. X. Naz S. Altaf M. M. Khan L. U.. (2022). Melatonin improves drought stress tolerance of tomato by modulating plant growth, root architecture, photosynthesis, and antioxidant defense system. Antioxidants 11 (2), 309. doi: 10.3390/antiox11020309 35204192 PMC8868175

[B2] Chen J. S. Lei N. F. Liu Q. (2011). Defense signaling among interconnected ramets of a rhizomatous clonal plant, induced by jasmonic-acid application. Acta Oecologica 37 (4), 355–360. doi: 10.1016/j.actao.2011.04.002

[B3] Chen Y. E. Cui J. M. Li G. X. Yuan M. Zhang Z. W. Yuan S.. (2016). Effect of salicylic acid on the antioxidant system and photosystem II in wheat seedlings. Biol. Plantrum 60 (1), 139–147. doi: 10.1007/s10535-015-0564-4

[B4] Corcuera L. Gil-Pelegrin E. Notivol E. (2011). Intraspecific variation in *Pinus Pinaster* PSII photochemical efficiency in response to winter stress and freezing temperatures. PloS One 6 (12), e28772. doi: 10.1371/journal.pone.0028772 22220195 PMC3248426

[B5] Frost C. J. Mescher M. C. Dervinis C. Davis J. M. Carlson J. E. De Moraes C. M. (2008). Priming defense genes and metabolites in hybrid poplar by the green leaf volatile cis-3-hexenyl acetate. New Phytol. 180 (3), 722–734. doi: 10.1111/j.1469-8137.2008.02599.x 18721163

[B6] Genty B. Briantais J. M. Baker N. R. (1989). The relationship between the quantum yield of photosynthetic electron transport and quenching of chlorophyll flourescence. Biochimica et Biophysica Acta(BBA)-General Subjects. 990 (1), 87–92. doi: 10.1015/s0304-4165(89)80016-9

[B7] Gomez S. Latzel V. Verhulst Y. M. Stuefer J. F. (2007). Costs and benefits of induced resistance in a clonal plant network. Oecologia 153 (4), 921–930. doi: 10.1007/s00442-007-0792-1 17609982 PMC2039789

[B8] Gomez S. Onoda Y. Ossipov V. Stuefer J. F. (2008). Systemic induced resistance: a risk-spreading strategy in clonal plant networks? New Phytol. 179 (4), 1142–1153. doi: 10.1111/j.1469-8137.2008.02542.x 18627496

[B9] Gomez S. Stuefer J. F. (2006). Members only: induced systemic resistance to herbivory in a clonal plant network. Oecologia 147 (3), 461–468. doi: 10.1007/s00442-005-0293-z 16333642

[B10] Gomez S. Van Dijk W. Stuefer J. F. (2010). Timing of induced resistance in a clonal plant network. Plant Biol. 12 (3), 512–517. doi: 10.1111/j.1438-8677.2009.00234.x 20522188

[B11] He L. X. Xiao X. Zhang X. M. Jin Y. Pu Z. H. Lei N. F.. (2021). Clonal fragments of stoloniferous invasive plants benefit more from stolon storage than their congeneric native species. Flora 281, 151877. doi: 10.1016/j.flora.2021.151877

[B12] He L. Yu L. Li B. Du N. Guo S. (2018). The effect of exogenous calcium on cucumber fruit quality, photosynthesis, chlorophyll fluorescence, and fast chlorophyll fluorescence during the fruiting period under hypoxic stress. BMC Plant Biol. 18 (1), 180. doi: 10.1186/s12870-018-1393-3 30180797 PMC6122546

[B13] Hu X. J. Chen D. Lynne Mclntyre C. Fernanda Dreccer M. Zhang Z. B. Drenth J.. (2018). Heat shock factor C2a serves as a proactive mechanism for heat protection in developing grains in wheat *via* an ABA-mediated regulatory pathway. Plant Cell Environ. 41 (1), 79–98. doi: 10.1111/pce.12957 28370204

[B14] Hunziker P. Lambertz S. K. Weber K. Crocoll C. Halkier B. A. Schulz A. (2021). Herbivore feeding preference corroborates optimal defense theory for specialized metabolites within plants. Proc. Natl. Acad. Sci. United States America 118 (47), 6. doi: 10.1073/pnas.2111977118 PMC8617496 34795057

[B15] Jelinkova H. Tremblay F. Desrochers A. (2012). Herbivore-simulated induction of defenses in clonal networks of trembling aspen (*Populus tremuloides*). Tree Physiol. 32 (11), 1348–1356. doi: 10.1093/treephys/tps094 23065192

[B16] Jia H. Lu C. (2003). Effects of abscisic acid on photoinhibition in maize plants. Plant Sci. 165 (6), 1403–1410. doi: 10.1016/j.plantsci.2003.08.004

[B17] Kaur G. Asthir B. (2020). Impact of exogenously applied ABA on proline metabolism conferring drought and salinity stress tolerance in wheat genotypes. Cereal Res. Commun. 48 (3), 309–315. doi: 10.1007/s42976-020-00041-0

[B18] Koubek T. Herben T. (2007). Effect of systemic diseases on clonal integration: modelling approach. Evolutionary Ecol. 22 (3), 449–460. doi: 10.1007/s10682-007-9219-z

[B19] Li K. N. Chen J. S. Wei Q. Li Q. Lei N. F. (2018). Effects of transgenerational plasticity on morphological and physiological properties of stoloniferous herb *centella asiatica* subjected to high/low light. Front. Plant Sci. 9. doi: 10.3389/fpls.2018.01640 PMC6246981 30487805

[B20] Lievens L. Pollier J. Goossens A. Beyaert R. Staal J. (2017). Abscisic acid as pathogen effector and immune regulator. Front. Plant Sci. 8. doi: 10.3389/fpls.2017.00587 PMC5395610 28469630

[B21] Liu X. Li Q. Yue M. Zhang X. Zhang R. Zhang B.. (2015). Nitric oxide is involved in integration of UV-B absorbing compounds among parts of clonal plants under a heterogeneous UV-B environment. Physiologia Plantarum 155 (2), 180–191. doi: 10.1111/ppl.12313 25424287

[B22] Lucas J. A. Garcia-Cristobal J. Bonilla A. Ramos B. Gutierrez-Manero J. (2014). Beneficial rhizobacteria from rice rhizosphere confers high protection against biotic and abiotic stress inducing systemic resistance in rice seedlings. Plant Physiol. Biochem. 82, 44–53. doi: 10.1016/j.plaphy.2014.05.007 24907524

[B23] Ma X. Ma F. Mi Y. Ma Y. Shu H. (2008). Morphological and physiological responses of two contrasting Malus species to exogenous abscisic acid application. Plant Growth Regul. 56 (1), 77–87. doi: 10.1007/s10725-008-9287-2

[B24] Miao R. Yuan W. Wang Y. Garcia-Maquilon I. Dang X. Li Y.. (2021). Low ABA concentration promotes root growth and hydrotropism through relief of ABA INSENSITIVE 1-mediated inhibition of plasma membrane H^+^ -ATPase 2. Sci. Adv. 7 (12), 4113. doi: 10.1126/sciadv.abd4113 PMC7968848 33731345

[B25] Osakabe Y. Yamaguchi-Shinozaki K. Shinozaki K. Tran L. P. (2014). ABA control of plant macroelement membrane transport systems in response to water deficit and high salinity. New Phytol. 202 (1), 35–49. doi: 10.1111/nph.12613 24283512

[B26] Qamer Z. Chaudhary M. T. Du X. Hinze L. Azhar M. T. (2021). Review of oxidative stress and antioxidative defense mechanisms in *Gossypium hirsutum* L. @ in response to extreme abiotic conditions. J. Cotton Res. 4 (1), 1–9. doi: 10.1186/s42397-021-00086-4

[B27] Ryu C. M. Farag M. A. Hu C. H. Reddy M. S. Kloepper J. W. Pare P. W. (2004). Bacterial volatiles induce systemic resistance in *Arabidopsis* . Plant Physiol. 134 (3), 1017–1026. doi: 10.1104/pp.103.026583 14976231 PMC389924

[B28] Sachdev S. Ansari S. A. Ansari M. I. Fujita M. Hasanuzzaman M. (2021). Abiotic stress and reactive oxygen species: generation, signaling, and defense mechanisms. Antioxidants (Basel) 10 (2), 277. doi: 10.3390/antiox10020277 33670123 PMC7916865

[B29] Sharifi R. Ryu C. M. (2021). Social networking in crop plants: Wired and wireless cross-plant communications. Plant Cell Environ. 44 (4), 1095–1110. doi: 10.1111/pce.13966 33274469 PMC8049059

[B30] Singh J. Thakur J. K. (2018). Photosynthesis and abiotic stress in plants. Biotic Abiotic Stress Tolerance Plants 27–46. doi: 10.1007/978-981-10-9029-5_2

[B31] Sperdouli I. Moustakas M. (2011). Differential response of photosystem II photochemistry in young and mature leaves of *Arabidopsis thaliana* to the onset of drought stress. Acta Physiologiae Plantarum 34 (4), 1267–1276. doi: 10.1007/s11738-011-0920-8

[B32] Sreenivasulu N. Harshavardhan V. T. Govind G. Seiler C. Kohli A. (2012). Contrapuntal role of ABA: does it mediate stress tolerance or plant growth retardation under long-term drought stress? Gene 506 (2), 265–273. doi: 10.1016/j.gene.2012.06.076 22771691

[B33] Srivastava M. K. Li C. Nong Q. Li Y. (2009). Effect of exogenous ABA application on chlorophyll fluorescence in sugarcane under water stress conditions. Guangxi Agric Sci. Guangxi Agric. Sci. 40 (11), 1411–1417.

[B34] Stuefer J. F. Gómez S. Mölken T. V. (2005). Clonal integration beyond resource sharing: implications for defence signalling and disease transmission in clonal plant networks. Evolutionary Ecol. 18 (5-6), 647–667. doi: 10.1007/s10682-004-5148-2

[B35] Su J. Yang L. Zhu Q. Wu H. He Y. Liu Y.. (2018). Active photosynthetic inhibition mediated by MPK3/MPK6 is critical to effector-triggered immunity. PloS Biol. 16 (5), e2004122. doi: 10.1371/journal.pbio.2004122 29723186 PMC5953503

[B36] Travaglia C. Reinoso H. Cohen A. Luna C. Tommasino E. Castillo C.. (2010). Exogenous ABA increases yield in field-grown wheat with moderate water restriction. J. Plant Growth Regul. 29 (3), 366–374. doi: 10.1007/s00344-010-9147-y

[B37] Turakainen M. Hartikainen H. Sarjala T. Seppänen M. M. (2008). Impact of selenium enrichment on seed potato tubers. Agric. Food Sci. 17 (3), 278–288. doi: 10.2137/145960608786118802

[B38] Turan Ö. Ekmekçi Y. (2010). Activities of photosystem II and antioxidant enzymes in chickpea (*Cicer arietinum* L.) cultivars exposed to chilling temperatures. Acta Physiologiae Plantarum 33 (1), 67–78. doi: 10.1007/s11738-010-0517-7

[B39] Underwood N. C. (1998). The timing of induced resistance and induced susceptibility in the soybean-Mexican bean beetle system. Oecologia 114, 376–381. doi: 10.1007/s004420050460 28307781

[B40] van Hulten M. Pelser M. Van Loon L. C. Pieterse C. M. Ton J. (2006). Costs and benefits of priming for defense in *Arabidopsis* . Proc. Natl. Acad. Sci. United States America 14 (103), 5602–5607. doi: 10.1073/pnas.0510213103 PMC1459400 16565218

[B41] Wei Q. Li Q. Jin Y. Wu S. L. Fan L. H. Lei N. F.. (2019). Transportation or sharing of stress signals among interconnected ramets improves systemic resistance of clonal networks to water stress. Funct. Plant Biol. 46 (7), 613–623. doi: 10.1071/FP18232 31010459

[B42] Wilson S. Ruban A. V. (2020). Enhanced NPQ affects long-term acclimation in the spring ephemeral Berteroa incana. Biochim. Biophys. Acta Bioenergetics 1861 (4), 148014. doi: 10.1016/j.bbabio.2019.03.005 30880080

[B43] Yoshida T. Christmann A. Yamaguchi-Shinozaki K. Grill E. Fernie A. R. (2019). Revisiting the basal role of ABA - roles outside of stress. Trends Plant Sci. 24 (7), 625–635. doi: 10.1016/j.tplants.2019.04.008 31153771

[B44] Yoshida T. Fujita Y. Sayama H. Kidokoro S. Maruyama K. Mizoi J.. (2010). AREB1, AREB2, and ABF3 are master transcription factors that cooperatively regulate ABRE-dependent ABA signaling involved in drought stress tolerance and require ABA for full activation. Plant J. 61 (4), 672–685. doi: 10.1111/j.1365-313X.2009.04092.x 19947981

[B45] Zhang J. Jia W. Yang J. Ismail A. M. (2006). Role of ABA in integrating plant responses to drought and salt stresses. Field Crops Res. 97 (1), 111–119. doi: 10.1016/j.fcr.2005.08.018

[B46] Zhu S. Q. Chen M. W. Ji B. H. Jiao D. M. Liang J. S. (2011). Roles of xanthophylls and exogenous ABA in protection against NaCl-induced photodamage in rice (*Oryza sativa* L) and cabbage (*Brassica campestris*). J. Exp. Bot. 62 (13), 4617–4625. doi: 10.1093/jxb/err170 21642236

[B47] Zou J. J. Wei F. J. Wang C. Wu J. J. Ratnasekera D. Liu W. X.. (2010). *Arabidopsis* calcium-dependent protein kinase CPK10 functions in abscisic acid- and Ca^2+^-mediated stomatal regulation in response to drought stress. Plant Physiol. 154 (3), 1232–1243. doi: 10.1104/pp.110.157545 20805328 PMC2971602

